# The Autism and Angelman Syndrome Protein Ube3A/E6AP: The Gene, E3 Ligase Ubiquitination Targets and Neurobiological Functions

**DOI:** 10.3389/fnmol.2019.00109

**Published:** 2019-04-30

**Authors:** Natasha Khatri, Heng-Ye Man

**Affiliations:** ^1^Department of Biology, Boston University, Boston, MA, United States; ^2^Department of Pharmacology and Experimental Therapeutics, Boston University School of Medicine, Boston, MA, United States

**Keywords:** neurodevelopement, UBE3A (E6AP), autism (ASD), Angelman syndrome (AS), ubiquitination, dendritic pruning, synaptic plasiticty

## Abstract

UBE3A is a gene implicated in neurodevelopmental disorders. The protein product of UBE3A is the E3 ligase E6-associated protein (E6AP), and its expression in the brain is uniquely regulated *via* genetic imprinting. Loss of E6AP expression leads to the development of Angelman syndrome (AS), clinically characterized by lack of speech, abnormal motor development, and the presence of seizures. Conversely, copy number variations (CNVs) that result in the overexpression of E6AP are strongly associated with the development of autism spectrum disorders (ASDs), defined by decreased communication, impaired social interest, and increased repetitive behavior. In this review article, we focus on the neurobiological function of Ube3A/E6AP. As an E3 ligase, many functional target proteins of E6AP have been discovered, including p53, Arc, Ephexin5, and SK2. On a neuronal level, E6AP is widely expressed within the cell, including dendritic arbors, spines, and the nucleus. E6AP regulates neuronal morphological maturation and plays an important role in synaptic plasticity and cortical development. These molecular findings provide insight into our understanding of the molecular events underlying AS and ASDs.

## Introduction

The human brain consists of 86 billion neurons, which are connected *via* trillions of synapses (Azevedo et al., 2009). The proper development of neurons and their connections, therefore, is critical for normal brain function. The establishment of brain structure and cortical layers begins during prenatal neuronal development, when neurons produced in the ventricular zone migrate radially out into the developing neocortex to form six distinct layers (Huang, 2009). After initial migration, neurons undergo extensive morphological change to form specific synaptic connections with target neurons *via* axon formation and dendritic arbor elaboration. Synaptic formation and refinement occur during prenatal and early postnatal periods in an activity-dependent manner, and brain circuitry can continue to be modified into adolescence and early adulthood. Disruption in any of these developmental processes can cause abnormalities in overall brain connectivity and lead to neurodevelopmental disorders.

Autism spectrum disorders (ASDs) are a heterogeneous class of neurodevelopmental disorders characterized by three main behavioral traits: impaired social interactions, lack of communication, and increased repetitive behaviors (Levy et al., 2009). However, these core clinical symptoms are often accompanied by other symptoms and disorders. Developmental comorbidities may include cognitive and intellectual disability, language deficits, attention problems, hyperactivity, and motor delays (Newschaffer et al., 2007; Levy et al., 2009). Psychiatric and related behavioral comorbidities include anxiety, depression, obsessive-compulsive disorder, defiant and aggressive behavior, and self-injurious behavior (Hartley et al., 2008; Simonoff et al., 2008). Other common comorbid features are seizures and epilepsy, gastrointestinal difficulties, and sleep disruption (Limoges et al., 2005; Rapin and Tuchman, 2008; Nikolov et al., 2009).

The genetic basis of ASDs is highly heterogeneous, as hundreds of different genes have been implicated in their cause. Interestingly, most of the genes show expression profiles at the stage of early development, and their functionalities share strong enrichment in cell adhesion and mobility, cytoskeleton regulation, synapse formation and kinase signaling (Pinto et al., 2010; Gilbert and Man, 2017). These ASD genes include FMR1, LIS1, MECP2, PTEN, SHANK1/2/3, TAOK2, TSC1/2, Neuroligins, Neurexins, KIAA2022/KIDLIA (Gilbert and Man, 2016) and UBE3A/E6-associated protein (E6AP). Pathological studies of ASD patients have revealed neurodevelopmental defects such as abnormal brain growth, impaired neuron morphology and brain cytoarchitecture, along with impaired synapse formation (Chen et al., 2015). The vast genetic landscape of ASDs and the resulting variability in pathology and causative pathways have made studying and treating ASDs a great challenge.

## Genomic Imprinting and Regulation of UBE3A/E6AP

**Figure 1:**
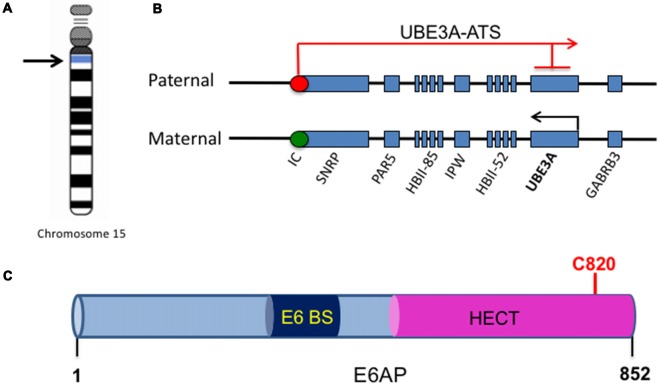
UBE3A imprinting and E6-associated protein (E6AP) structure. **(A)** The UBE3A gene is located on chromosome 15 within the region of 15q11-15q13. **(B)** Within the chromosome region 15q11-q13, the gene *UBE3A* is maternally imprinted in the brain. A paternally expressed antisense transcript (UBE3A-ATS) initiates at the unmethylated imprinting center (IC, red circle) of the paternal allele and overlaps UBE3A, silencing the paternal expression of the gene in the brain. This imprinting results in expression solely from the maternal allele in the brain, as the maternal imprinting center is methylated (IC, green circle). **(C)** E6AP is an 862-amino acid protein with a C-terminal homology to E6AP C-terminus (HECT) E3 ligase domain. It also contains a binding site for the human papillomavirus type 16 (HPV16) protein E6 (E6 BS). The catalytic cysteine of E6AP is located at C820 (red).

One of the major genes implicated in ASDs is the Ubiquitin Protein Ligase E3A, *UBE3A*, the gene that encodes E6AP, a protein that is expressed in an imprinted manner in the brain. From here onwards, *UBE3A* will refer to the gene, and E6AP will refer to its protein product. Genomic imprinting marks the parental origin of chromosomal subregions and results in allele-specific differences in DNA methylation, transcription, and replication. Within the chromosome region 15q11-q13, the gene *UBE3A* is imprinted specifically in the brain, resulting in maternal expression of E6AP in human fetal brain and adult cortex, while the paternal copy is silenced (Figures 1A,B; Rougeulle et al., 1997; Vu and Hoffman, 1997). Similar imprinting in *UBE3A* also exists in rats and mice (Albrecht et al., 1997). Although the mechanism of tissue-specific *UBE3A* imprinting is not fully understood, its expression in general has been found to be mediated by the presence of an antisense transcript, *UBE3A-ATS*, which is paternally expressed (Rougeulle et al., 1998). *UBE3A-ATS* is a ~460-kb noncoding RNA that initiates in the 15q11-q13 region of the paternal allele and overlaps *UBE3A*, silencing the paternal expression of the gene in the brain (Figure 1B; Runte et al., 2001). Similarly, the murine *Ube3A-ATS* is also observed to be paternal-specific and restricted to the brain (Chamberlain and Brannan, 2001). Interestingly, *UBE3A* imprinting occurs only in neurons in the brain; both alleles are active in glial cells and other peripheral tissues (Yamasaki et al., 2003).

The *UBE3A* gene encodes three potential E6AP protein isoforms generated by differential splicing (Yamamoto et al., 1997). The coding region of E6AP is 2,700 bp long and consists of 10 exons, encoding for 865 amino acids (Huibregtse et al., 1993a). Isoforms 2 and 3 have an additional 20 and 23 amino acids, respectively, at their amino-terminus. Although isoforms 2/3 have similar E3 ligase catalytic function, it is unknown whether the variable amino terminus could account for differential ubiquitination substrate specificity (Yamamoto et al., 1997). Interestingly, a recent study reported that Ube3A isoform 1 RNA is encoded by a truncated sequence of the gene and does not include the E3 catalytic domain sequence (Valluy et al., 2015). Interestingly, while it is not detectably translated into protein, its expression is involved in the regulation of dendritic complexity and spine maturation. It was suggested that Ube3A1 RNA might be a target for microRNA miR-134, providing a novel protein expression-independent function of Ube3A (Valluy et al., 2015).

Neuronal activity can alter expression of E6AP (Greer et al., 2010). Specifically, expression of E6AP mRNA in cultured neurons is increased by either membrane depolarization or glutamate receptor activation, while blocking activity with NMDA receptor, sodium channel, or AMPA receptor (AMPAR) inhibitors decreased E6AP mRNA expression. In addition, E6AP expression is induced in response to environmental stimuli that trigger experience-dependent synaptic development, as shown in mice that received an enriched environment compared to those in a standard cage. This increase was found to be regulated by the binding of the activity-regulated transcription factor myocyte enhancer factor 2 (MEF2) to *UBE3A* promoters 1 and 3 (Greer et al., 2010). Interestingly, MEF2 has previously been shown to control synapse development and regulates a number of genes that have been implicated in ASDs (Flavell et al., 2006; Morrow et al., 2008). Further involvement of synaptic activity on the levels of E6AP was reported in another study (Filonova et al., 2014). Levels of nuclear and cytoplasmic E6AP were increased after neuronal depolarization in primary neuron culture, and upregulation of E6AP was observed in mice with an E6AP-YFP reporter following fear conditioning (Filonova et al., 2014). Additionally, a lack of E6AP led to deficits in the increased activity-dependent phosphorylation of the kinase ERK1/2, a process that is important in synaptic plasticity and memory formation (Thomas and Huganir, 2004; Filonova et al., 2014). These studies suggest that E6AP levels are regulated by synaptic activity and that loss of experience and activity-dependent induction of E6AP expression during postnatal development may contribute to ASDs.

## Role of *UBE3A*/E6AP Gene Dosage and Protein Levels in Angelman Syndrome and ASD

Proper gene dosage of *UBE3A* is crucial to normal brain development, as evidenced by the neurodevelopmental disorders associated with deletions, mutations, and copy number variations (CNVs) of *UBE3A*. Angelman syndrome (AS) was characterized behaviorally by Harry Angelman to consist of “puppet”-like behavior, a distinctive feature of AS (Angelman, 1965). AS manifests itself as a severe developmental delay with a virtual absence of speech and abnormal gait (Williams et al., 1995). In addition, patients exhibit coordination difficulties, a contagiously happy demeanor, prominent laughing, tongue protrusion, and a seizure disorder (Williams et al., 1995). Some characteristics of AS may be seen on the spectrum of autistic features, such as impaired communication, absence of speech, attentional deficits, hyperactivity, feeding and sleeping problems, and delay of motor development (Williams et al., 2001, 2006).

AS is primarily caused by deletions and mutations in *UBE3A*, and its specific genetic causes are differentiated by five molecular classes (Lossie et al., 2001; Clayton-Smith and Laan, 2003). Class I accounts for 65%–70% of AS cases and is caused by a *de novo* deletion of the maternal chromosome 15q11-q13, causing a loss of all E6AP expression in the brain (Clayton-Smith and Laan, 2003). Class II patients have uniparental disomy (UPD) for chromosome 15 and therefore fail to inherit a maternal copy of *UBE3A* (Clayton-Smith and Laan, 2003). Class III patients are those without deletions or UPD, but with abnormal methylation of the chromosome 15 maternal allele, resulting in a defect in maternal expression (Reis et al., 1994). Class IV patients are those who have mutations within *UBE3A* (Kishino et al., 1997; Matsuura et al., 1997). Point mutations in AS patients have been found throughout the entire coding region with clusters in exon 9, which contains the E6AP homology to E6AP C-terminus (HECT) domain. Many mutations, including frameshift, nonsense, and splice mutations, have been found to be located within the region encoding the catalytic cleft between the two lobes of the HECT domain (Cooper et al., 2004). Finally, Class V patients are designated as those with a clinical phenotype of AS with no chromosome 15 abnormality (Lossie et al., 2001).

A potential treatment for the imprinting defects in AS may be to unsilence the dormant paternal allele in neurons and restore E6AP expression despite the loss of maternal expression (Mabb et al., 2011). Indeed, an unbiased, high throughput screen in neurons from AS mice lead to the discovery of 12 topoisomerase I inhibitors and four topoisomerase II inhibitors that unsilence the paternal *UBE3A* allele (Huang et al., 2011). One of the drugs found, topotecan, upregulated levels of active E6AP by downregulating the paternal *UBE3A-ATS*. Expression of the paternal *UBE3A* allele was unsilenced by topotecan in the hippocampus, neocortex, striatum, cerebellum, and spinal cord, suggesting that silencing the *UBE3A-ATS* and reactivating paternal expression of E6AP may serve as a potential therapeutic strategy for patients with AS (Huang et al., 2011). Similarly, it has been shown that expression of a truncated Ube3A-ATS unsilenced paternal E6AP and was able to ameliorate behavioral deficits in AS mice (Meng et al., 2013). More importantly, reactivation of Ube3A expression in a Cre-dependent manner during early development was shown to rescue behavioral phenotypes, while reinstatement during adulthood improved the electrophysiological deficits in layer 5 pyramidal neurons (Silva-Santos et al., 2015; Rotaru et al., 2018). Consistent with the beneficial effect of Ube3A reinstatement, a study in AS mice showed that restoring Ube3A expression in GABAergic neurons suppressed the occurrence of epileptic activity (Gu et al., 2019).

ASDs, on the other hand, are caused by CNVs in the *UBE3A* gene. Individuals with an additional maternal copy of *UBE3A* (dup15), due to duplication of the 15q11.2–11.3 chromosomal region, and those with two extra copies from an isodicentric chromosome 15 (idic15) both display autism penetrance, with the two extra copies resulting in a more severe phenotype (Borgatti et al., 2001; Hogart et al., 2010). Consistent with the imprinted expression of *UBE3A*, ASDs arise from maternally, but not paternally, derived 15q11-q13 duplications (Cook et al., 1997). These genetic studies suggest a role for *UBE3A* dosage in neuronal development.

## Animal Models of Angelman Syndrome and *UBE3A*-Dependent ASD

The *UBE3A* maternal deficient mouse model of AS (Ube3A^m−/p+^), in which a deletion mutation in exon 2 of *UBE3A* inhibits maternal expression of the gene, successfully captures many of the classical features associated with AS and is the most widely used AS mouse model (Jiang et al., 1998). Ube3A^m−/p+^ mice exhibit reduced brain weight, ataxia, motor impairments, abnormal EEG, and audiogenic seizures. These mice also display context-dependent learning and memory impairments, and deficits in hippocampal long-term potentiation (LTP; Jiang et al., 1998). Importantly, the degree of behavioral EEG activity phenotypes varies based on the genetic background of Ube3A^m−/p+^ mice (Born et al., 2017). Mice on a C57BL/6J background displayed robust behavioral impairments, such as decreased activity and marble burying, increased anxiety, and altered novel object recognition, along with spontaneous EEG polyspikes and increased spectral power. Mice on a 129 background performed poorly on a wire hand test and contextual fear conditions and had a lower seizure threshold. Mice on a F1 hybrid background showed milder behavioral impairments, and fewer EEG polyspikes and spectral power alterations, raising the awareness that small genetic variances in mice could lead to discrepancies in observed phenotypes (Born et al., 2017).

Increased gene dosage of *UBE3A* has been modeled in mice to mimic the *UBE3A* CNVs in ASDs. The Ube3A 2X transgenic (Tg) mouse model exhibits a tripling of the normal Ube3A gene dosage in neurons, replicating idic15 in patients with autism (Smith et al., 2011). Ube3A 2XTg mice show typical autistic behavioral deficits, including impaired social behavior, as measured by social preference tests, decreased communication, measured by vocalizations, and increased repetitive behavior, shown by excessive grooming. In addition, recordings in hippocampal slices showed reduced strength in excitatory synaptic transmission, both in frequency and amplitude, suggesting that E6AP may regulate glutamate transmission at both pre- and post-synaptic sites (Smith et al., 2011). More recently, it was found that E6AP regulates levels of the synaptic protein Cbln1 in these mice and that recurrent seizures led to increased *Cbln1* mRNA in the ventral tegmental area (VTA; Krishnan et al., 2017). Importantly, restoring *Cbln1* levels in neurons of the VTA reversed the impaired sociability behavioral phenotype of Ube3A 2X mice, suggesting that Cbln1 levels play a key role in E6AP-dependent ASD behavior (Krishnan et al., 2017). Interestingly, the activity of the VTA, specifically the VTA-to-nucleus accumbens (NAc) dopaminergic projections, is sufficient and necessary to control key features of social behavior, as demonstrated by optogenetic modulation of the pathway (Gunaydin et al., 2014).

## Brain and Cellular Distribution of E6AP

Knowledge of the imprinting and expression pattern of E6AP in the brain has come from studying various brain regions and tissues in the maternally-deficient Ube3A^m−/p+^ mice. It has been shown that maternal E6AP is expressed in the hippocampus, hypothalamus, olfactory bulb, cerebral cortex, striatum, midbrain, and cerebellum (Gustin et al., 2010). Expression is seen primarily in neurons, both excitatory and inhibitory neurons (Gustin et al., 2010). Within neurons, E6AP is enriched in the nucleus and dendrites in mouse brain tissue (Dindot et al., 2008). In cultured hippocampal neurons, E6AP also localizes to the nucleus and to presynaptic and postsynaptic compartments (Dindot et al., 2008).

The expression of imprinted E6AP in the brain also seems to be temporally regulated. To study imprinting and the resulting expression, mouse models lacking either the paternal or maternal copy of *UBE3A* have been utilized (UBE3A^m+/−p−^ or UBE3A^m−/p+^). In the visual cortex, low levels of expression of paternal E6AP remain during early postnatal development at postnatal day 6 (P6), indicating that the paternal allele is not completely silenced at this stage (Sato and Stryker, 2010). Conversely, expression of E6AP at later developmental time points, around P27–P29, stem primarily from the maternal allele expression (Sato and Stryker, 2010). Although paternal E6AP expression becomes undetectable in neurons beyond the first postnatal week in mice, maternal E6AP is expressed throughout postnatal development and into adulthood (Judson et al., 2014). However, this imprinting may not occur similarly throughout the brain. Although cortical lysates show residual expression of E6AP in Ube3A^m−/p+^ mice at birth that is diminished by adulthood, presumably from expression of the paternal allele, sub-cortical and cerebellar tissues express levels of E6AP at birth that are comparable to those in adult mice (Grier et al., 2015). Late-onset silencing of paternal Ube3A has also been observed in induced pluripotent stem cells (iPSCs) derived from an AS patient (Stanurova et al., 2016). These findings suggest that in AS mice and AS patients, normal development of neurons may occur while paternal E6AP expression remains, but developmental deficits begin to arise as paternal expression diminishes and the lack of maternal expression leads to a complete loss of E6AP function in the brain. The timing of this imprinting pattern suggests that deficits in AS may occur during a postnatal critical period of experience-dependent neuronal development.

## E6AP Structure and Function

E6AP was first discovered as the ubiquitin protein ligase involved in the degradation of the tumor repressor p53 (Scheffner et al., 1993). Human papillomavirus type 16 (HPV16) viral infections are associated with malignant lesions leading to cervical cancer and encode the oncoprotein E6 (zur Hausen, 1991). The E6 protein leads to degradation of the tumor repressor p53 in cells infected with HPV16, which was mediated by the involvement of a 100 kDa protein (Huibregtse et al., 1991). Indeed, that protein was termed E6AP and was found to be a necessary component in the ubiquitination and degradation of p53 in cancer cells (Scheffner et al., 1993). The binding region for E6 is localized to the N-terminal of E6AP, from amino acid 391–408, while the p53 binding domain consists of 500 amino acids. Additionally, the last 84 amino acids of E6AP were required for p53 degradation (Huibregtse et al., 1993b). The COOH-terminal 350 amino acids of E6AP comprise the HECT domain, a region shared by several E3 ligases structurally similar to E6AP, and this domain is required for the ubiquitination function of E6AP (Huibregtse et al., 1993a, 1995; Figure 1C). Furthermore, the catalytic active site of E6AP is localized to a cysteine at position 833, as mutating this cysteine to alanine renders the E3 ligase unable to form a thioester bond with ubiquitin (Scheffner et al., 1995). E6AP can also self-ubiquitinate in HPV16-positive cells and mediate its own degradation (Kao et al., 2000). This requires the binding of E6 to E6AP and is mediated by the intramolecular transfer of ubiquitin from the active cysteine site of E6AP to one of its own lysine residues, possibly acting as a multimer in order to achieve self-ubiquitination (Kao et al., 2000).

Crystal structure of E6AP showed that the HECT domain consists of two lobes that pack loosely across a small interface and are connected by a three-residue hinge (residues 738–740). The larger NH_2_-terminal lobe of the HECT domain (residues 495–737) has a mostly α-helical structure, while the smaller COOH-terminal lobe (residues 741–852) has an α/β structure and contains the catalytic Cys^820^ (Huang et al., 1999). Notably, many E6AP mutations in AS patients are located in the HECT domain and around the catalytic site (Nawaz et al., 1999; Cooper et al., 2004). Several AS mutations that affect E6AP substrate ubiquitination inhibit the E3 ligase from forming a thioester bond with ubiquitin (Cooper et al., 2004). In addition, an autism-linked missense mutation disrupts E6AP phosphorylation by protein kinase A (PKA) at residue T485 and leads to an enhancement of its activity towards other substrates (Yi et al., 2015). Thus, there is a strong link between the E3 ligase function of E6AP and its involvement in neurodevelopmental disorders, suggesting that E3 ligase function is essential to the role of E6AP in normal brain development.

Another function of E6AP has been discovered as coactivator for the nuclear hormone receptor superfamily. Nuclear hormone receptors are ligand-induced transcription factors that require coactivators to achieve optimal function (Shibata et al., 1997). Coactivators enhance receptor function by acting as a bridge between DNA-bound receptors and basal transcription factors (Chen et al., 1997). E6AP contains a nuclear localization signal that allows it to be localized to the nucleus, and three LXXL motifs, which are important for receptor interaction (Hatakeyama et al., 1997; Heery et al., 1997). E6AP was found to interact with the liganded form of the progesterone receptor and increase its transcriptional activity (Nawaz et al., 1999). Interestingly, its function as a receptor coactivator is independent from its function as a ubiquitin protein ligase. However, further evidence is needed to support the contribution of the nuclear hormone receptor in Ube3A-dependent AS and ASD pathogenesis.

## Primary Ubiquitination Targets of E6AP E3 Ligase

**Figure 2:**
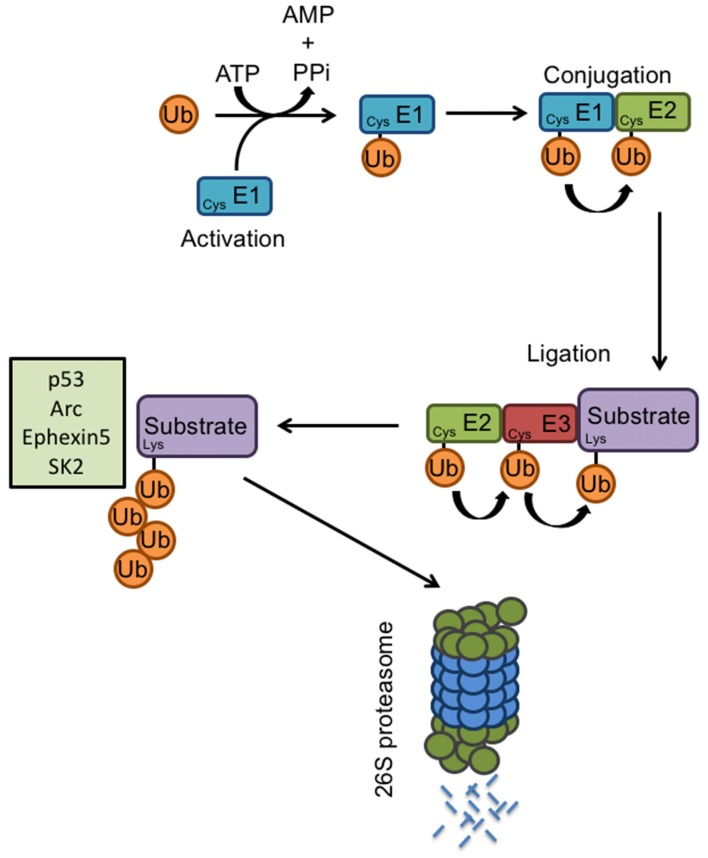
E3 ligases and the ubiquitin proteasome system. Proteasome-mediated degradation of proteins involves the addition of ubiquitin to specific target molecules followed by their trafficking to the proteasome for degradation into small peptides and amino acids. This process occurs *via* coordinated actions of three classes of enzymes: E1, E2, and E3. The ubiquitin-activating enzyme E1, activates the free ubiquitin in an ATP-dependent manner. The conjugating enzyme E2 then carries the transfer of the activated ubiquitin, and a substrate-specific E3 ligase attaches the ubiquitin molecule to a target protein. Once a ubiquitin molecule has attached to a protein, another ubiquitin can be attached to an internal lysine residue of the first ubiquitin, and this can go on to form a polyubiquitin chain on the target protein. Known E6AP targets include p53, Arc, Ephexin5, and SK2. Polyubiquitination tags a protein substrate for degradation and causes it to be trafficked to the 26S proteasome.

The proteolysis of specific substrates *via* the ubiquitin-proteasome pathway (UPS) is essential to neuronal development and synaptic plasticity (Hegde and DiAntonio, 2002). Proteasome-mediated degradation of proteins involves the addition of ubiquitin to specific target molecules followed by their trafficking to the proteasome for degradation into small peptides and amino acids. This process occurs *via* coordinated actions of three classes of enzymes: E1, E2, and E3 (Figure 2). E1, the ubiquitin-activating enzyme, activates the free ubiquitin in an ATP-dependent manner. The conjugating enzyme E2 then carries the transfer of the activated ubiquitin, and a substrate-specific E3 ligase attaches the ubiquitin molecule to a target protein. Once a ubiquitin molecule has attached to a protein, another ubiquitin can be attached to an internal lysine residue of the first ubiquitin, and this can go on to form a polyubiquitin chain on the target protein. Polyubiquitination tags a protein substrate for degradation and causes it to be trafficked to the 26S proteasome (Hegde, 2004). At the synapse, ubiquitination can modulate neurotransmitter receptors, as well as components of the postsynaptic density (Ehlers, 2003). The UPS also plays an important role in cell growth, neurite extension, structural remodeling, and synaptic formation and plasticity (d’Azzo et al., 2005; Hurley et al., 2006; Nandi et al., 2006; Shearwin-Whyatt et al., 2006; Segref and Hoppe, 2009). Impairments in ubiquitin-mediated protein degradation can, therefore, lead to deficits in neuronal development and the maintenance of synaptic connections.

As the primary function of E6AP is that of an E3 ligase and its function is mediated *via* protein ubiquitination, it is critical to identify its downstream targets. To date, several E6AP ubiquitination targets have been identified, including the tumor suppressor p53, the PDZ-containing protein Scribble, the transcriptional repressor NFX1-91, the DNA-repair protein HHR23A, the AMPAR-trafficking regulator Arc, the RhoA guanine nucleotide exchange factor Ephexin5, and the small-conductance potassium channel SK2.

### The Tumor Repressor P53

The tumor repressor p53 was one of the first ubiquitination targets discovered for E6AP in the context of viral infection by HPV16. Although most of the studies on the interaction between E6AP and p53 have focused on its role in cancer, p53 has also been studied in the context of AS. In mice maternally deficient for E6AP, increased cytoplasmic p53 was found in Purkinje and hippocampal cells compared to wild type (WT) mice (Jiang et al., 1998). No differences in p53 transcripts were found between the mice, suggesting that the change in p53 levels was due to a posttranscriptional effect. In addition, increased p53 immunoreactivity was found in Purkinje cells of a patient with clinical diagnosis of AS, suggesting that E6AP can regulate levels of p53 in the absence of the E6 viral protein (Jiang et al., 1998). Although these *UBE3A^m-/p+^* mice display impaired contextual learning and hippocampal LTP, it is unclear whether the changes in p53 contribute to the behavioral phenotype of AS mice. Furthermore, how the ubiquitination of p53 by E6AP affects neuronal developmental or morphology in the context of AS or ASD remains to be studied.

### The Human Homolog of the Yeast DNA Report Protein Rad 23 (HHR23A)

One of the substrates identified with the normal cellular function of E6AP, as opposed to its role in cancer cells, is HHR23A, the human homolog of the yeast DNA repair protein Rad23 (Kumar et al., 1999). HHR23A levels are increased in response to DNA damage, and its levels are also regulated in a cell-cycle dependent manner, with specific degradation occurring during the S phase. HHR23A binds E6AP and is ubiquitinated *in vitro* in a cell-cycle and E6AP-dependent manner, which is enhanced with the overexpression of WT E6AP, but not the E3 ligase mutant E6AP C833A (Kumar et al., 1999). Although this study provides important information on the cellular function of E6AP in DNA repair and cell cycle progression *via* regulation of HHR23A levels, the role of this ubiquitination target has not been studied in the context of AS and ASD.

### The Synaptic Protein Arc

Arc is an immediate early gene protein with its expression tightly regulated by neuronal activity (Link et al., 1995; Lyford et al., 1995). Arc has been shown to play important roles in AMPAR trafficking and synaptic plasticity (Chowdhury et al., 2006; Rial Verde et al., 2006; Shepherd et al., 2006). In elucidating the molecular mechanisms underlying AS, Arc was shown to be a target for E6AP-mediated ubiquitination (Greer et al., 2010). Arc regulates the trafficking of AMPARs at the synapse by accelerating endocytosis and reducing surface expression (Chowdhury et al., 2006). In mouse brain extracts, Arc was associated with E6AP. Increased expression of E6AP, but not the E3 ligase mutant E6AP C833A, led to increased Arc ubiquitination (Greer et al., 2010). Under the conditions of increased neuronal activity, either by kainic acid or an enriched environment, Ube3A^m−/p+^ AS mice have higher levels of Arc than WT animals. Neurons transfected with E6AP shRNA have reduced levels of the AMPAR subunit GluA1, which was caused by increased endocytosis of GluA1 and resulted in decreased miniature excitatory postsynaptic currents (mEPSCs). The reduction in GluA1 was mediated by E6AP-dependent ubiquitination of Arc, and Ube3A^m−/p+^ mice also had decreased levels of AMPARs (Greer et al., 2010). Further supporting this work was the finding that seizure-like activity in the AS mouse model could be attenuated by reducing Arc expression (Mandel-Brehm et al., 2015). However, the ubiquitination-dependent degradation of Arc by E6AP has been challenged. Another study demonstrated a lack of interaction between full-length Arc and E6AP, and Arc ubiquitination and its total protein levels seemed not affected by increased E6AP expression (Kuhnle et al., 2013). Furthermore, they showed that down-regulation of E6AP expression stimulates estradiol-induced transcription of the Arc gene, suggesting that Arc protein levels are controlled by E6AP at the transcriptional rather than post-translational level (Kuhnle et al., 2013).

### The RhoA Guanine Nucleotide Exchange Factor Ephexin5

EphB receptors are expressed on developing axons and dendrites and regulate actin cytoskeleton remodeling critical for excitatory synapse development *via* binding to their ligand EphrinBs and the subsequent activation of guanine nucleotide exchange factors (GEFs; Klein, 2009). Activation of EphBs in hippocampal neurons leads to an increase in dendritic spines and functional excitatory synapses, whereas disruption of EphB function leads to defects in spine morphogenesis and a decrease in excitatory synapse number (Ethell et al., 2001; Henkemeyer et al., 2003; Penzes et al., 2003; Kayser et al., 2006). Ephexin5, a RhoA GEF expressed in the brain, negatively regulates excitatory synapse development until EphrinB binding to the EphB receptor tyrosine kinase triggers Ephexin5 (E5) phosphorylation, ubiquitination, and degradation. The degradation of E5 promotes EphB-dependent excitatory synapse development and was found to be mediated by E6AP (Margolis et al., 2010). A Ube3A binding domain (UBD) sequence, corresponding to the E6AP-binding sequence of HHR23A, was identified in Ephexin5. Furthermore, immunoprecipitation showed binding between E6AP and E5. E5 levels were decreased in the presence of E6AP, but not the E3 ligase mutant E6AP C833A, and E5 degradation was attenuated by shRNA-mediated knockdown of E6AP (Margolis et al., 2010). Additionally, E5 levels in the brains of Ube3A^m−/p+^ mice were significantly higher and E5 ubiquitination levels were reduced, supporting the role of E6AP in mediating E5 degradation by ubiquitination and potentially regulating excitatory synapse formation (Margolis et al., 2010).

### Small-Conductance Potassium Channel 2 (SK2)

Small-conductance potassium channels (SKs) are involved in synaptic transmission by contributing to the hyperpolarization after an action potential or repolarization after EPSCs (Adelman et al., 2012). In hippocampal neurons, synaptic SK channels become active upon NMDAR activation, leading to membrane repolarization and thus suppression of the NMDAR activity, a function that is important in regulating neuronal excitability for LTP, a well-studied form of synaptic plasticity important for learning and memory (Nicoll and Malenka, 1999; Malinow and Malenka, 2002). In turn, LTP induction regulates levels of synaptic SK2 by triggering endocytosis (Lin et al., 2008). Recently, it was discovered that E6AP ubiquitinates SK2 and facilitates its internalization (Sun et al., 2015). Specifically, ubiquitination by E6AP was found to occur at the C-terminal K506/K514/K550 residues of SK2. Furthermore, synaptic SK2 levels were increased in the hippocampus of Ube3A^m−/p+^ mice, along with decreased ubiquitination of SK2. This resulted in impaired synaptic plasticity and decreased NMDAR function, suggesting that E6AP can modulate synaptic plasticity by regulating SK2 channel levels *via* ubiquitination and endocytosis (Sun et al., 2015).

Additional new targets have been continuously discovered in recent studies, such as the mTORC1 regulating protein p18 and the inhibitor of apoptosis XIAP (Khatri et al., 2018; Sun et al., 2018). Ube3A has been shown to regulate mTORC1 signaling by targeting the Ragulator complex subunit p18 for proteasomal degradation (Sun et al., 2018). Ube3A deficiency increases lysosomal localization of p18 and the Ragulator complex, and leads to increased mTORC1 activity and eventual improvement in dendritic spine maturation and learning performance. This study provides a novel mechanism by which Ube3A can modulate synaptic activity *via* its function as an E3 ligase (Sun et al., 2018). In our recent study, we have identified XIAP as a Ube3A target for ubiquitination and degradation, which is involved in aberrant dendritic arborization in Ube3A-dependent ASD (Khatri et al., 2018).

## Role of *UBE3A*/E6AP in Neurite Growth and Neuronal Maturation

The signs and symptoms of ASDs often appear before 3 years of life, a time window when social, emotional, and cognitive skills are developing (Walsh et al., 2008). This period correlates with a development phase of brain architecture, including the generation of new neurons, dendritic growth, synaptogenesis, neuron circuit formation, and experience-dependent remodeling (Fox et al., 2010).

A growing number of studies have revealed a role for E6AP in neuron structural development. Alterations in E6AP levels have led to changes in dendritic and spine morphology. Ube3A^m−/p+^ AS mice have dendritic spines with inconsistent morphology, including variability in spine neck length and head size (Dindot et al., 2008). Hippocampal dendritic spines were lower in density and shorter in length in Ube3A^m−/p+^ mice than in WT mice (Dindot et al., 2008). When E6AP was downregulated in mice *via* *in utero* electroporation of shRNA, changes in the polarity of dendrites was observed (Miao et al., 2013). Specifically, at P7, the orientation property of the apical dendrite relative to the line perpendicular to the pial surface in layer 2/3 neurons was impaired in neurons with E6AP shRNA. The length of the apical dendrite was also reduced compared to control neurons, both in cortical and hippocampal neurons. These deficits were attributed to the regulation of Golgi apparatus distribution; control neurons had Golgi enriched within the apical dendrite, whereas E6AP shRNA-transfected neurons had Golgi clustered near the nucleus. Interestingly, these deficits were rescued by the overexpression of shRNA-resistant E6AP isoform 2, the primary E6AP isoform expressed in the brain from embryonic to adult stages in mice (Miao et al., 2013). Furthermore, stunted apical dendrites and decreased dendritic polarity were observed in Ube3A^m−/p+^ mice (Miao et al., 2013). Supporting the role for Golgi dysfunction in AS, another study reported structural disruption of cisternal swelling of the Golgi apparatus in Ube3A^m−/p+^ cortical neurons (Condon et al., 2013). Golgi were found to be severely under-acidified, leading to osmotic swelling, and resulting in a marked reduction in protein sialylation, a process dependent on Golgi pH (Condon et al., 2013).

Morphological deficits have also been found in *Drosophila* with a loss of dUBE3A, the homolog for Ube3A/E6AP. In the absence of dUBE3A, the number of terminal dendritic branches in class IV da sensory neurons was reduced (Lu et al., 2009). Da neurons in dUBE3A-null neurons fail to completely prune their dendrites during early metamorphosis, suggesting a pruning defect. Strikingly, overexpression of dUBE3A in da neurons decreased dendritic branching, implicating an important role for the dosage of E6AP in neuronal development (Lu et al., 2009).

Abnormalities in dopamine signaling have been found in Ube3A^m−/p+^ mice. Although the number of dopaminergic cells and dopamine synthesis are normal in these AS mice, increased dopamine release was observed in the mesolimbic pathway, while the nigrostriatal pathway exhibited decreased dopamine release (Riday et al., 2012). Decreased GABA co-release was also found as a result of E6AP loss from tyrosine hydroxylase-expressing dopaminergic neurons in the VTA, leading to enhanced reward-seeking behavior (Berrios et al., 2016). Interestingly, clinical administration of levodopa (L-DOPA) in a small number of AS patients dramatically improved resting tremor and rigidity symptoms (Harbord, 2001). These studies suggest that although defects in the dopaminergic pathway may not account for all neurodevelopmental effects of E6AP loss, it may be involved in causing some of the symptoms arising from dopaminergic signaling.

More recently, our own work showed that in cultured hippocampal neurons, ASD-related overexpression of Ube3A led to a drastic remodeling of dendritic arborization, mainly by a reduction in dendrite number and length (Khatri et al., 2018). This remodeling effect was mediated by the ubiquitination and degradation of XIAP by E6AP, which led to the activation of caspase-3 and subsequent cleavage of microtubules (Khatri et al., 2018). Strikingly, the Ube3A 2X ASD mouse model displayed a similar reduction in dendritic branching in cortical neurons, along with decreased XIAP levels, increased caspase-3 activation, and elevated levels of tubulin cleavage. Spine morphology was also affected by overexpression of Ube3A, as decreased spine density and increased spine length were observed both *in vitro* and *in vivo* (Khatri et al., 2018). These findings reveal an important mechanism for increased Ube3A gene dosage in ASD-related neurodevelopmental alterations, and further implicate the role of Ube3A/E6AP in neuronal morphology growth and maturation. Interestingly, in some studies, reduced Ube3A expression also leads to a reduction in dendritic arborization (Miao et al., 2013; Valluy et al., 2015), suggesting that an opposite change in Ube3A gene dosage may trigger distinct signaling cascades, which nevertheless cause similar changes in dendritic development.

## The Role of E6AP in Neural and Synaptic Plasticity

Ube3A^m−/p+^ mice were shown to have impaired experience-dependent synaptic plasticity in the visual cortex (Yashiro et al., 2009). Specifically, Ube3A^m−/p+^ do not exhibit ocular dominance plasticity induced by monocular deprivation, and visual cortex neurons show decreased mEPSCs in response to visual experience (Yashiro et al., 2009). Cortical circuitry and retinotopic maps form normally, with no obvious defects seen in cell density and overall cortical development in the visual cortex. However, spine density in the basal dendrites of the Layer V visual cortex neurons is reduced in Ube3A^m−/p+^ mice (Sato and Stryker, 2010). The time for ocular dominance formation represents a critical period for experience-dependent visual cortex maturation, and UBE3A maternal allele expression is increased during this critical period, suggesting that E6AP plays a role in postnatal experience-dependent neuronal development (Sato and Stryker, 2010). Interestingly, in Ube3A^m−/p+^ mice, reinstatement of E6AP expression at birth and at 3 weeks of age was able to rescue motor deficits, while reinstatement in adults failed to show rescue effects, suggesting the existence of a developmental time window with high sensitivity to E6AP activities (Silva-Santos et al., 2015).

E6AP has also been implicated in the expression of LTP. In the hippocampus of Ube3A^m−/p+^ mice, increased levels of inhibitory phosphorylation at Thr305 of CaMKII were found, thereby decreasing the activity of the protein, which is important in the induction of LTP (Weeber et al., 2003; Lisman et al., 2012). This change in CaMKII function was thought to be responsible for some of the learning impairments in Ube3A^m−/p+^ mice, as the behavioral and learning deficits were reversed when a mutation was introduced to block the inhibitory phosphorylation of CaMKII (van Woerden et al., 2007). E6AP was also shown to modulate NMDAR-mediated synaptic plasticity by ubiquitinating and internalizing SK2 channels (Sun et al., 2015).

An alteration in the excitatory/inhibitory (E/I) balance is increasingly considered a key feature in ASD pathogenesis (Nelson and Valakh, 2015). Indeed, a loss of E6AP leads to an E/I imbalance in the brains of Ube3A^m−/p+^ mice, which may contribute to seizure susceptibility in AS (Wallace et al., 2012). Inhibitory GABAergic drive onto layer 2/3 pyramidal neurons in the visual cortex is decreased with loss of maternal E6AP, which arises from an accumulation of clathrin-coated vesicles at inhibitory axon terminals in interneurons. However, excitatory interneuron input is not affected, suggesting that the E/I balance is neuron-specific (Wallace et al., 2012). Furthermore, selective loss of E6AP in GABAergic neurons causes AS-like neocortical EEG patterns, enhancing seizure susceptibility, and leads to presynaptic accumulation of clathrin-coated vesicles, whereas specific glutamatergic loss has no effect on EEG patterns (Judson et al., 2016). Decreased tonic inhibition has also been found in cerebellar granule cells of E6AP-deficient mice (Egawa et al., 2012). E6AP was found to control the degradation of the GABA transporter 1 (GAT1) in cerebellar granule cells, leading to an increase in GAT1 with loss of E6AP and resulting in decreased GABA concentrations in the extrasynaptic space. Additionally, treatment of a selective GABA_A_ receptor agonist improved the firing properties of cerebellar cells in brain slices and reduced cerebellar ataxia in Ube3A^m−/p+^ mice, further supporting the role of neuron-specific effects of E6AP loss resulting in the manifestation of various behavioral phenotypes in AS (Egawa et al., 2012). More recently, it was shown that increased sensitivity to seizures in AS mice was attributed to Ube3A deletion in GABAergic but not glutamatergic neurons, and the epileptic behavior was rescued by reinstatement of Ube3A in the GABAergic cells during development (Gu et al., 2019).

## Conclusion

Loss of function of Ube3A/E6AP results in the manifestation of AS, whereas duplication and triplication of the gene cause autism, suggesting the sensitivity of neurodevelopmental processes to the E6AP dosage. As an E3 ligase, the discovery of specific ubiquitination targets and their neuronal function is critical to the understanding of AS and E6AP-dependent ASDs. Many studies using mouse models have elucidated a mechanism by which E6AP alters dendrite and spine formation, and synaptic plasticity during an experience-dependent critical window in brain development. E6AP targets may be widely distributed in a neuron including the nucleus, neurites and the spines, but the exact subcellular location of E6AP activity execution remains less clear. Future work will elucidate the specific contribution of cellular and molecular alterations to the aberrant brain development and behavioral phenotype in AS and ASD.

## Author Contributions

NK and HYM wrote the manuscript.

## Conflict of Interest Statement

The authors declare that the research was conducted in the absence of any commercial or financial relationships that could be construed as a potential conflict of interest.
